# Effect of pre-hatch incubator lights on the ontogeny of CNS opsins and photoreceptors in the Pekin duck

**DOI:** 10.1016/j.psj.2022.101699

**Published:** 2022-01-10

**Authors:** Anna Vostrizansky, Andrew Barce, Zoe Gum, Daniel J. Shafer, Debbie Jeffrey, Gregory S. Fraley, Phillip D. Rivera

**Affiliations:** ⁎ Hope College, Department of Biology, Holland, MI 49423, USA; † Maple Leaf Farms, Inc., Leesburg, IN, USA

**Keywords:** Pekin duck, ontogeny, photoreceptors, retina, deep brain photoreceptors, *OPN4M*

## Abstract

The Pekin duck is a valuable agricultural commodity globally and in the United States. Pekin ducks are seasonal breeders; they are sensitive to light and thus, research on the neuroendocrine and behavioral responses are needed to maximize production and to improve their welfare. There is compelling evidence that specific wavelengths of light may adversely alter the growth and welfare of meat (grow out) ducks. However, despite a birds’ dependence upon light, in commercial poultry hatcheries, incubators almost exclusively hold eggs in the dark. Therefore, our objective was to determine the effects of lighting on the expression of retina photoreceptors (**RPs**) and deep brain photoreceptors (**DBPs**) during duck embryological development. Two groups of ducks were raised with and without light over 21 d from egg laying, embryonic day 0. Brain and retinal tissues were collected at embryonic days 3, 7, 11, 16, and 21 of a 24 d incubation period. qRT-PCR was performed on RPs (*OPN1LW, OPN2SW, OPN1SW, MAFA, RHO*, and *RBP3*) and the DBP *OPN4M* from retinal and brain samples, respectively. We find that the presence and absence of light during pre-hatch incubation, had no influence on the expression of any retinal photoreceptor. However, a late embryological increase in DBP *OPN4M* expression was observed. Taken together, the impact of light during pre-hatch incubation does not impact the overall post-hatch production. However, future directions should explore how *OPN4M* pre-hatch activation impacts Pekin duck post-hatch development and growth.

## Highlights

• Incubated eggs with and without light had no effect on post-hatch production.
• Light does not influence the ontogeny of retinal rod and cone photoreceptors.
• Brain OPN4 mRNA is increased the later stages of embryonic development.

## INTRODUCTION

The Pekin duck is a major economic commodity worldwide, the third largest meat bird and second largest poultry export in the United States, producing nearly 31 million birds per year. Losses in the duck industry occur from embryonic mortality and a lack of neonatal viability (Cherry and Morris, 2008). These losses can have a profound impact on the economics of duck production and negatively impact the ability for supply to meet worldwide demand for duck meat. One possible solution is the addition of lights to incubators in order to strengthen growth and vitality. Several experiments have been conducted in the past using multiple colors of monochromatic lights, such as red, blue, white, and green, however, these studies have produced mixed results (Follett and Pearce-Kelly, 1991; Zhang et al., 2014; Sabuncuoğlu et al., 2018). Some research has focused on the use of lights in the incubator to improve post-hatch performance (Reinert and Wilson, 1996; Wilson and Reinert, 1996; Li and Kuenzel, 2008; Pérez et al., 2016; Mishra et al., 2017). There are as many conflicting findings as there are corroborative and many of these differences could be explained by differences in incubators. Although there are many interesting findings, a much better understanding of lighting and photoreceptor biology of the developing embryo is needed for this exciting field.

Follett and Pearce-Kelly (1991) suggested that red light may penetrate living tissue more successfully than blue light, since it has a lower energy and power of penetration than blue or green spectra. In contrast, Sabuncuoglu et al. (2018) found Japanese quail eggs incubated in green and blue light show no significant differences in incubation traits, post-hatch growth, and behavioral traits. Other labs have found that the implementation of green light during development promotes muscle growth in broiler chickens (Zhang et al., 2014). A possible reason for these mixed results is that in order to respond to light physiologically, an organism must express photoreceptors (Reinert and Wilson, 1996; Wilson and Reinert, 1996; Li and Kuenzel, 2008; Pérez et al., 2016; Mishra et al., 2017). Although the pineal gland regulates diurnal rhythms, the major site for multi-spectra PRs are the retina and diencephalon of the brain (Kuenzel et al., 2015).

Non-image forming (**NIF**) retinal ganglion cells have been identified in the retina of chickens. These NIF have been shown to be intrinsically photosensitive retinal ganglion cells (Ana Contin et al., 2006; Contín et al., 2010). The ipRGCs have been shown in both sighted and blind chickens to perceive light and transmit this information to the brain via an independent neuronal pathway than that of retinal visual PRs (Valdez et al., 2009) and by that of diencephalic PRs (Valdez et al., 2013). PRs in the diencephalon are referred to as deep brain photoreceptors (**DBPs**) and are known to be responsible for stimulating photoreceptivity and gonadal recruitment in birds and mice (Scott et al., 1986; Wilson, 2001; Kang and Kuenzel, 2015), in addition to the duck (Haas et al., 2017; Potter et al., 2018). In mammals, photoreceptivity includes the activity of rods and cones within the retina, as well as NIF, intrinsically photosensitive retinal ganglion cells (**ipRGCs**; for review see Hatori and Panda, 2010). NIF ipRGCs regulate environmental light responses, including circadian entrainment (Panda et al., 2002, 2003) and the pupillary light reflex (Heaton, 1971). These cells are also known to activate diencephalic nuclei, such as the suprachiasmatic nucleus, and in turn affect melatonin secretion from the pineal gland (for review, see Wagner et al., 2008). In birds, the removal of these retinal cells does not affect seasonal changes in the hypothalamic-pituitary-gonadal axis (**HPG**; Oliver and Baylé, 1982). However, if light is prevented from penetrating the skull, a loss in photoresponsiveness leads to gonadal regression in the house sparrow (Menaker et al., 1970) and lizard (Underwood and Menaker, 1970). Hence, in birds there is compelling evidence that photoresponsiveness is mediated—at least in part—by non-retinal neurons that express photosensitive chemicals. These neurons have been referred to as DBPs (reviewed in Kuenzel et al., 2015). Activation of DBPs has been suggested to be a prerequisite for the increase in hypothalamic-pituitary-thyroid axis activity (Kuenzel, 1993; Wilson and Reinert, 1996; Wilson, 2001; Li and Kuenzel, 2008; Kang and Kuenzel, 2015). Indeed, numerous studies have reported a causative link between thyroid hormone activity and development (Péczely et al., 1979; Harvey et al., 1982; Reinert and Wilson, 1996; Schew et al., 1996; Výboh et al., 1996; Wilson and Reinert, 1996, 1999). DBPs also appear to be evolutionarily conserved, with melanopsin (OPN4M) receptors being found in various mammals that are responsive to blue-specific light wavelengths (Iyilikci et al., 2009; Bailes and Lucas, 2013; Tsunematsu et al., 2013; Ramos et al., 2014; Walmsley et al., 2015). Interestingly, with the photoperiodic responsiveness of melanopsin (El Halawani et al., 2009; Kang et al., 2010), DBPs may play a critical role in normal development. However, the ontogeny of DBP expression is unknown in the Pekin duck.

Lighting has profound effects on the physiology and welfare of all poultry species, and the Pekin duck is no exception. One of the challenges to implement new lighting systems or programs is that bird vision is fundamentally different from human vision. For example, birds can see more colors, discriminate between colors better, process visual information faster, and integrate visual inputs from different parts of their lateralized visual fields. Additionally, the sensory physiology of the eye can change with age (Hodos et al., 1991; Porciatti et al., 1992) that can ultimately change visual perception. Furthermore, there is also evidence suggesting that the ambient light properties during development can alter the sensitivity of cone PRs in chickens (Hart, 2001). Consequently, any new lighting system ought to be developed relative to the visual system of the target species taking into account potential changes in eye development. Similar to DBPs, little is known about the ontogeny of retinal PRs in the duck.

The purpose of our study was to determine the ontogeny of PRs in the developing retina and diencephalon of the Pekin duck under dark and light conditions. To accomplish this goal, we utilized qRT-PCR to determine if incubation in dark vs. light had an impact on the expression patterns of retinal and brain photoreceptors. We hypothesized that incubation of eggs under light would increase the expression of PRs and improve the hatchability of eggs, compared to an absence of light.

## METHODS

### Animals and Housing

Standard commercial breeder strains of Pekin duck eggs were placed in one of 2 commercial incubators (Buckeye, Single Stage Incubator, Model, SS-112) at Maple Leaf Farms, Inc., (Leesburg, IN) one housed with “poultry” LEDs obtained from Once Innovation, Inc. (Agrishift; Plymouth, MN) and the other in the absence of light (dark). On extraction days, eggs were removed and embryos immediately removed for dissection. When eggs were removed from the dark incubators, all external lights were turned off during the extraction. Temperature gradients and humidity were maintained at industry standards. LED lights were placed on the walls of the incubators in such a way as to illuminate all trays similarly. Lights off was at 1800 h and lights on at 0600 h. All eggs were candled on incubation d 12, and numbers of clear eggs and early deads were recorded. On incubation d 23 (after the final day of extraction for our embryonic study) all eggs were put into a hatcher with either the same type or schedule of LED or kept dark as in their respective incubators. Light intensity data loggers (Hobo Inc.) were placed on the edges and center of the trays and recorded every 1 min throughout the length of the incubation period. In addition, light spectral make-up and photonic energy were measured using a spectrophotometer (USB5000UV, OceanOptics, Inc., Raleigh, NC). Supplemental Figure 1 illustrates representative recordings from both the data loggers and spectrophotometer. Production data (percent clear eggs, percent dead embryos, percent rotted eggs when moved to hatcher, post hatch wk 1 and 2 mortality, and final body and evisceration weight at production at 35 d) recorded for remaining eggs (n = 8,000 per treatment) in the incubators. Ducks were raised to processing weight (∼4 kg at 35 d) in separate pens within the same commercial barns using identical environmental and management conditions (Maple Leaf Farms, Inc., Leesburg, IN).

All lights in both the incubator and hatcher were from LED bulbs and the energy of all lights was normalized at the level of the duck eggs, as determined by spectrophotometry (USB5000UV, Ocean Optics, Inc). Eggs in the incubators are placed on a reciprocating tray, tilting to 45° to simulate rotation of eggs, thus all eggs spent 50% facing the light source and the other 50% of time facing 45° away from light source. Light recordings were made as the eggs were both facing and not facing the light source (Supplementary Figure 1). All study procedures were approved by the Hope College Animal Care and Use Committee following the Institutional Animal Care and Use Committee guidelines.

### Study Design and Sample Collection

Brain tissue samples from light or dark incubators were collected on d 3, 7, 11, 16, and 21 of incubation (extraction day, **ED**). Eggs were selected as those known to be anatomical hallmarks of visual system development (Fite and Bengston, 1989). Dark treatment samples were extracted 2 d prior to light treatment samples for all timepoints, but at the same embryonic age. Entire embryo (ED 3 and 7), retinal tissue (ED 11, 16, and 21), and brain tissue (ED 11, 16, and 21) were extracted from light and dark treatment groups (n = 10 treatment group/ED timepoint starting, with a final group range from 6 to 10 samples). Embryos from the dark incubator were removed from the egg and dissected under dark conditions. Supplementary Figure 2 illustrates the sampling milieu. Collection of the samples was performed at the hatchery, and placed into TRIzol (Thermo Fisher Scientific, 15596026, Waltham, MA). Samples were placed into TRIzol (200 μL dark 3 ED, 400 μL all 7, 11, and 16 retina ED, 500 μL 16 brain ED, and 800 μL 21 ED, respectively) at the time of dissection and were immediately frozen on dry ice.

### RNA and DNA Extraction, Quantification, and Purity Determination

Frozen samples (isolated cells) were thawed and incubated at room temperature (**RT**) for 5 min then homogenized in TRIzol. Samples were centrifuged at 11,800 rpm for 15 min a 4°C. Samples were spun and the supernatant was removed and placed inside the new tube. Depending on the initial volume of TRIzol used (200 μL, 400 μL, 500 μL, and 800 μL) either 80 μL, 100 μL, 160 μL, or 200 μL of chloroform, respectively (4:1), was added to the supernatant and vortexed for 2 min at 2,000 rpm on MixMate. Samples were then incubated at RT for 3 mins and then centrifuged at 11,800 rpm for 15 min at 4°C. A density gradient was created with which the top clear aqueous phase of RNA was separated into a fresh tube. Following this, 1 μL of Glycogen was added to the aqueous phase followed by an aqueous layer to isopropanol (1:1, isopropanol:TRIzol). Samples were vortexed for 1 min, incubated for 10 min at RT, and centrifuged at 11,800 rpm for 10 mins at 4°C. The supernatant was discarded carefully without disturbing RNA pellet.

Pellets were washed 2 times with 200 μL, 400 μL, 500 μL, or 800 μL of 75% ethanol (1:1, ethanol:TRIzol, ThermoFisher Scientific). Tubes were inverted on a clean Kimwipe and pellets were allowed to air dry until translucent/clear. Pellets were then resuspended in RNase-free water, based on pellet size and mixed carefully by flicking. Samples were placed on ice for a minimum of 10 min to mix and stored in −20°C freezer for short term (e.g., going to cDNA synthesis the same day) or −80°C freezer for long term. Preliminary RNA quantification and purity determination was done using a NanoDrop 2000 Spectrophotometer (Thermo Scientific, Wilmington, DE). Afterward, 1 uL of RNA sample was loaded on the spectrophotometer for measurement. RNA concentration was recorded at 260 nm wavelengths and RNA purity was determined by the 260/230 and 260/280 ratios. RNA was considered pure if 260/280 (RNA:protein contamination) ratio was in the range of 1.7 to 2.0, and 260/230 (RNA:Ethanol Contamination) was between 2.0 and 2.2. For all samples RNA was normalized to 100 ng/μL and stored in a −80 freezer for no more than 2 wk.

### qPCR

Primers (Table 1) were cross-referenced with NCBI BLAST using the ref seq genome of *Anas platyrhynchos* and analyzed with OligoAnalyzer to determine optimal free energies. Quantitative real-time PCR (**qPCR**) was carried out using Luna Universal One-Step RT-qPCR Kit (New England BioLabs Inc., Ipswich, MA, E3005X) and qPCR primers were designed in-house and purchased from Integrated DNA Technologies (Coralville, IA). The following PRs were selected for the study, and classified as retinal PRs from cones (e.g., *OPN1LW* [ascension, NM_205440.2], *OPN2SW* [ascension, NM_205517.2], *OPN1SW* [ascension, NM_205438.1]), rods (*MAFA* [ascension, XM_027452788.2], *RHO* [ascension, XM_005012054.4], and *RBP3* [ascension, NC_051777.1]), and DBPs (e.g., *OPN4M* [ascension, NM_001044653.1]). Developmental housekeeping genes (*GAPDH, SDHA, RSP13*) were averaged and used at every time point for all gene expression analyses. These genes were chosen as they do not differ statistically from one another across developmental time points (data not shown).

**Table 1:** Primer sequences.

Tissue	Gene	Forward	Reverse
Cones	*OPN1LW*	CGCCATCATCATCCTCTGCT	GACTCCGACTCCTTCTGCTG
	*OPN2SW*	TGACGAAGATGGTGGTGGTG	GATGACGGGGTTGTAGACGG
	*OPN1SW*	CTGAGCCCGTTCTGTGTCTT	GTTTCCATCCACCCCTCCTG
Rods	*MAFA*	CTACTGGATGTCGGGCTACC	TCGTCGGAAAAACGCTCCTC
	*RHO*	GCCAGTGCTTGTGCTTTTGA	GGGTGACCAATGGGGGAAAT
	*RBP3*	TGATCGTAGTACCTCCCGCA	ACAGCCCCAATGTCCACAAA
DBP	*OPN4M*	CTCGCCATAGAACATCCGCA	ACTGAACAGGCTACTCCCCTT
Housekeeping	*GAPDH*	GGTTGTCTCCTGCGACTTCA	TCCTTGGATGCCATGTGGAC
	*RPS13*	AAGAAAGGCCTGACTCCCTC	TGCCAGTAACAAAGCGAACC
	*SDHA*	GACACAGTGAAAGGCTCCGA	CTCCAGCTCTATCACGGCAG

### Plate Design

Each gene was assessed in duplicate using a MicroAmp Fast Optical 96-Well Reaction Plate, 0.1 mL (Applied Biosystems, Carlsbad, CA, 4346907). Mastermix was composed of 10 μL Luna blue reaction dye, 1 μL Luna WarmStart, 1.6 μL of primer, 5.4 μL of nuclease-free water, and 2 μL of template RNA. For gene expression analysis, a single sample was assayed in duplicate. Plates were sealed, and qPCR was run for 40 cycles with a cover temperature of 105.0°C.

### Statistical Analyses

qPCR data was analyzed with GraphPad PRISM version 8.1.2 (GraphPad Software, San Diego, CA) using a repeated measures (**RM**) mixed-effects analysis. Degrees of freedom for all statistical analyses were corrected by Greenhouse-Geisser method due to low n's per group. Post-hoc analyses were performed using Bonferroni's multiple comparisons test. For Figure 3B, a one-way ANOVA was performed on the combined data with a Brown-Forsythe and Welch's ANOVA to correct for within group variances. Post-hoc analysis using Dunnett's T3 multiple comparisons test was then performed. See Table 2 for all statistical analyses. All data are presented as means ± standard error of the means (**SEM**).

**Table 2:** Statistical analyses of all data.

Tissue	Gene	F	df	*P* value	Effect size (95% CI)
Cones	*OPN1LW*				
Repeated measures (RM) mixed-effects analysis	Time	0.64146	1.571, 26.31	*P* > 0.05	(−0.03107, 0.1733)
	Treatment	1.93	1, 67	*P* > 0.05	(−0.03107, 0.1733)
	Interaction	0.6051	4, 67	*P* > 0.05	(−0.03107, 0.1733)
	*OPN2SW*				
Repeated measures (RM) mixed-effects analysis	Time	1.003	1.774, 26.16	*P* > 0.05	(−0.01504, 0.1364)
	Treatment	2.834	1, 18	*P* > 0.05	(−0.01504, 0.1364)
	Interaction	1.113	4, 59	*P* > 0.05	(−0.01504, 0.1364)
	*OPN1SW*				
Repeated measures (RM) mixed-effects analysis	Time	0.7515	1.591, 30.24	*P* > 0.05	(−0.01618, 0.09182)
	Treatment	1.946	1, 76	*P* > 0.05	(−0.01618, 0.09182)
	Interaction	1.433	4, 76	*P* > 0.05	(−0.01618, 0.09182)
Rods	*MAFA*				
Repeated measures (RM) mixed-effects analysis	Time	0.9447	1.202, 23.13	*P* > 0.05	(−0.01599, 0.08133)
	Treatment	1.787	1, 77	*P* > 0.05	(−0.01599, 0.08133)
	Interaction	0.9028	4, 77	*P* > 0.05	(−0.01599, 0.08133)
	*RHO*				
Repeated measures (RM) mixed-effects analysis	Time	2.647	1.421, 27.36	*P* > 0.05	(−0.03162, 0.09325)
	Treatment	0.9658	1, 77	*P* > 0.05	(−0.03162, 0.09325)
	Interaction	0.3191	4, 77	*P* > 0.05	(−0.03162, 0.09325)
	*RBP3*				
Repeated measures (RM) mixed-effects analysis	Time	1.644	2.198, 34.07	*P* > 0.05	(0.001324, 0.1576)
	Treatment	4.132	1, 62	*P* < 0.05	(0.001324, 0.1576)
	Interaction	0.8269	4, 62	*P* > 0.05	(0.001324, 0.1576)
DBP	*OPN4M*				
Repeated measures (RM) mixed-effects analysis	Time	19.38	1.517, 26.17	*P* < 0.05	(−0.07750, 0.09074)
	Treatment	0.02465	1, 69	*P* > 0.05	(−0.07750, 0.09074)
	Interaction	2.512	4, 69	*P* < 0.05	(−0.07750, 0.09074)
Brown-Forsythe ANOVA	*OPN4M*	19.48	4, 33.17	*P* < 0.05	
Welch's ANOVA	*OPN4M*	13.96	4, 32.34	*P* < 0.05	

## RESULTS

### Production Data

**Figure 1:**
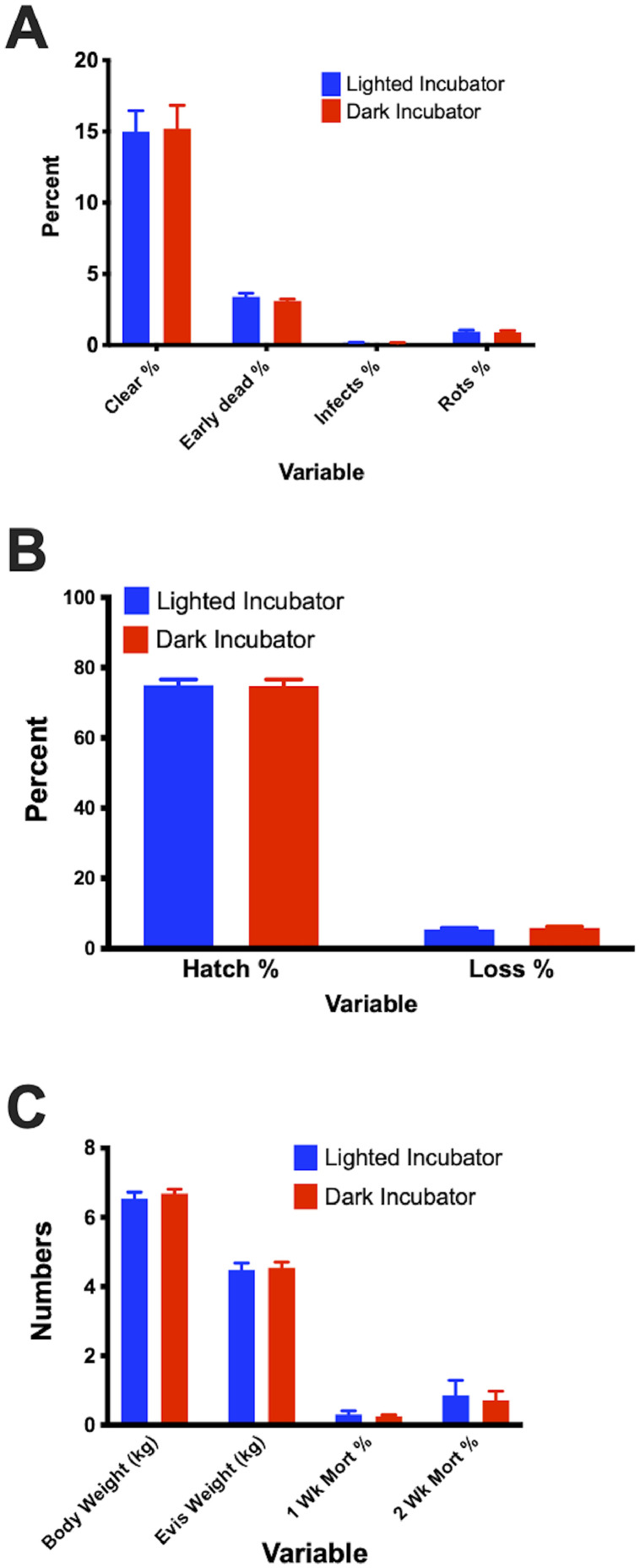
Production data. (A) Percent of clear eggs, early deads, infects, and rots. There was no difference in the number of early dead, infected, or rotted egg percentages between lighted and dark incubation. (B) Hatchability percentage. There was no difference in the percent of eggs hatched or the percentage of ducklings lost due to neonatal death between those raised in light and dark incubators. (C) Body weight (kg) of ducks at market weight (35 d), eviscerated carcass weight, and percent mortality at 1 and 2 wk of age. There was no difference in body weight or eviscerated carcass weight, week-old weight, and 2-wk old weight of birds that were incubated in light or dark incubators. Data are presented as mean ± SEM. Abbreviations: Evis, eviscerated; Mort, mortality; Wk, week.

No significant differences were observed in any of the hatchery or post-hatch production data between lighted and dark incubators. Figure 1 illustrates these data.

### Cone and Rod Photoreceptors

**Figure 2:**
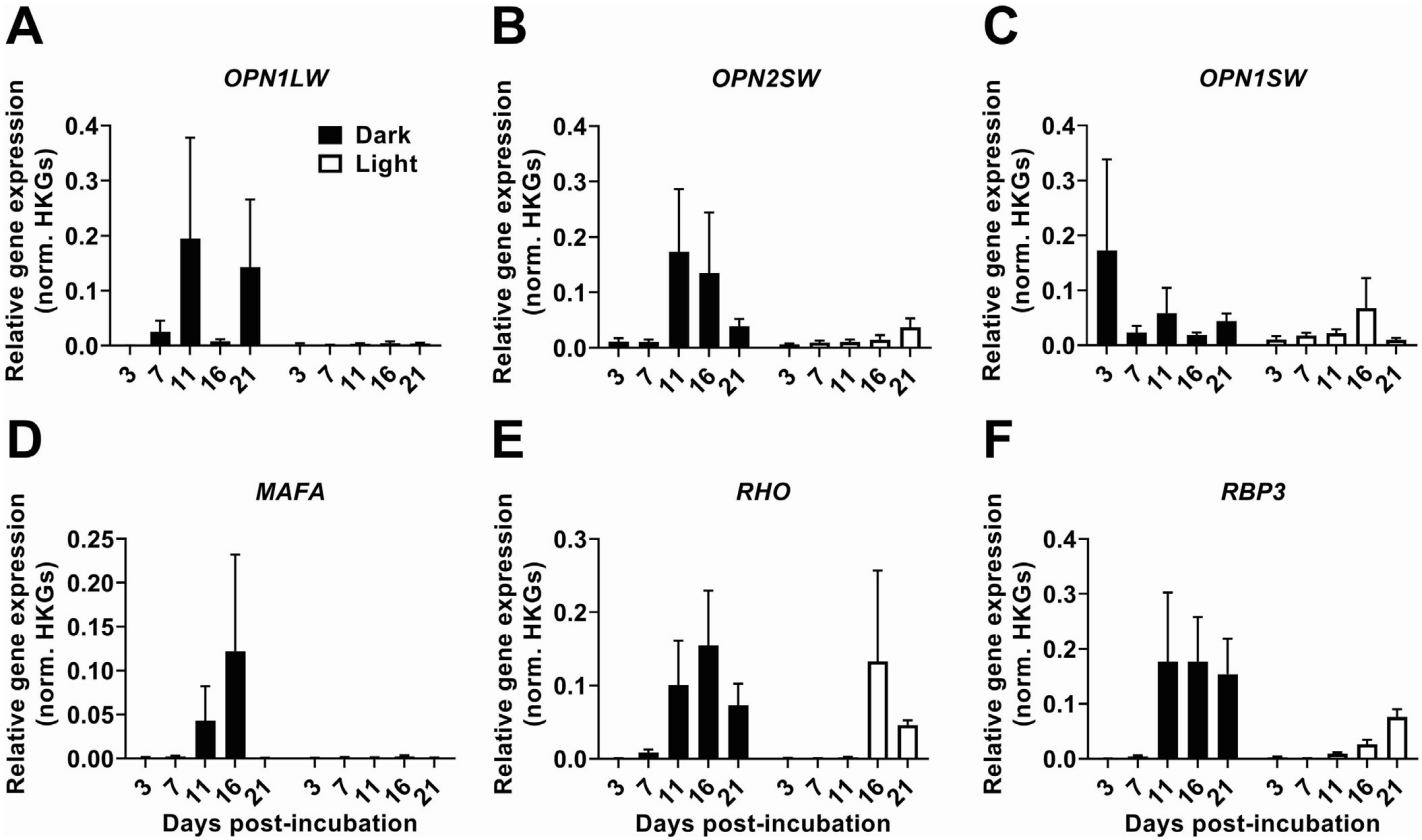
The absence of light does not significantly impact the relative gene expression of cones (OPN1LW, OPN2SW, and OPN1SW) and rods (MAFA, RHO, and RBP3) in the retina across a development. (A–E) No main effect of time or treatment was found using repeated measures mixed-effect ANOVA. (F) A main effect of Treatment (F(1,62) = 4.132, *P* < 0.05) was found. No main effect of Time (F(2.198,34.07) = 1.644, *P* > 0.05) nor interaction (F(4,62) = 0.8269, *P* > 0.05) was observed using a repeated measure mixed-effect ANOVA. Posthoc analysis did not show significant differences between dark and light treatment groups at any day postincubation. Data are presented as mean ± SEM. Abbreviation: HKGs, House keeping genes.

Previous studies have shown varying results using green light, as well as red and blue light incubators to aid in duck growth and development (Follett and Pearce-Kelly, 1991; Zhang et al., 2014; Sabuncuoğlu et al., 2018). However, it is unclear when duck embryos can perceive light. Therefore, we performed qPCR across timepoints examining cones (*OPN1LW, OPN2SW,* and *OPN1SW,* Figures 2A–2C) and rods (*MAFA*, RHO, RBP3, Figures 2D–2F) in the embryo and retina. For cones and rods, duck embryos received light or dark treatment, extracted at both 3 and 7 ED. Retina tissue also received light or dark treatment and were extracted at 11, 16, and 21 ED. Relative gene expression analysis using a repeated measure mixed-effect ANOVA revealed no significant interaction across development for all genes analyzed (Table 2). One exception was *RBP3* that showed no main effect of Time (F_(2.198, 34.01)_ = 1.644, *P* > 0.05), but did show a significant main effect of Treatment (F_(1, 62)_ = 4.132, *P* < 0.05), and no significant Time × Treatment (F_(4, 62)_ = 0.8269, *P* > 0.05; Figure 2F).

### Deep Brain Photoreceptor (OPN4)

**Figure 3:**
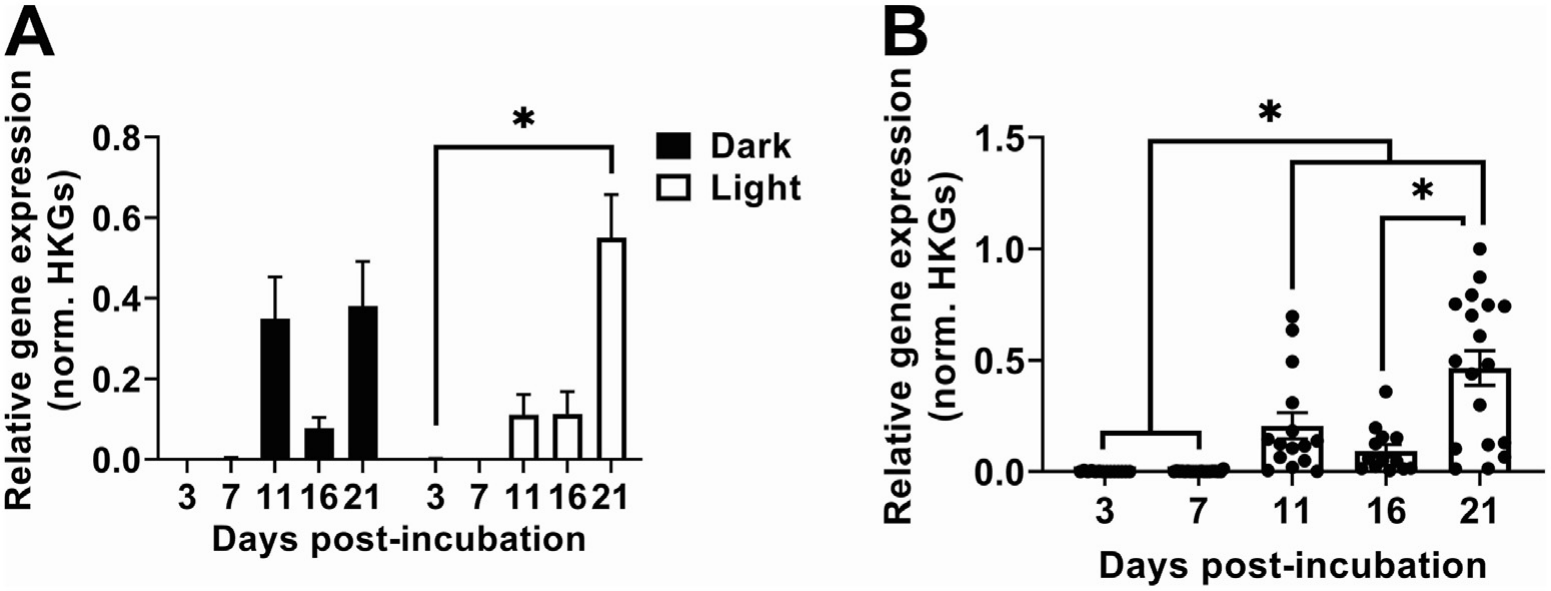
*OPN4M* gene expression increases during embryonic development. (A) A main effect of Time (F_(1.517, 26.17)_ = 19.38, *P* < 0.05) and a significant interaction between Time × Treatment (F_(4, 69)_ = 2.512, *P* < 0.05) was found. No main effect of Treatment (F_(1,69)_ = 0.02465, *P* > 0.05) was observed using a repeated measure mixed-effect ANOVA. (B) Light and Dark groups combined. Brown-Forsythe (F_(4, 33.17)_ = 19.48, *P* < 0.05) and Welch's (F_(4, 32.34)_ = 13.96, *P* < 0.05) ANOVA. Post-hoc using Dunnett's T3 multiple comparisons test. Data are presented as mean ± SEM. Abbreviation: HKGs, house keeping genes.

Previous studies have shown that after the removal of an eye (Menaker et al., 1970; Wilson et al, 1991), sensory activity was maintained in the duck, and lizard (Underwood et al., 1970), in non-retinal neurons known as DBP. In addition, opsin genes *OPN2* and *OPN5* and other vertebrate ancient opsin mRNA were not detected in the embryo when compared to the adult duck (Haas et al., 2017). Therefore, we examined melanopsin (*OPN4M*) to assess DBP gene expression across a developmental time course with and without light. Relative gene expression analysis of using a repeated measure mixed-effect ANOVA revealed a significant main effect of Time (F_(1.517, 26.17)_ = 19.38, *P* < 0.05) across development. *OPN4M* relative gene expression also showed a significant interaction between Time x Treatment (F_(4, 69)_ = 2.512, *P* < 0.05). There was no main effect of Treatment (F_(1,69_) = 0.02465, *P* > 0.05). Post-hoc analysis showed a significant difference between ED 3 compared to ED 21, only in the light treated group (*P* < 0.05, Figure 3A). With no significant differences observed between treatment groups, light and dark groups were combined and a Brown-Forsythe and Welch's ANOVA was performed across development to examine the ontogeny of OPN4M in the Pekin duck brain. Post-hoc analysis showed a significant decrease in OPN4M early development (ED3 and ED7) compared to older embryos (ED 11 and ED 21, *P* < 0.05). ED 16 was also observed to be significantly lower than ED21 (*P* < 0.05, Figure 3B), but not ED 1, 3, nor 11.

## DISCUSSION

The purpose of this study was to determine if lights in a commercial incubator could advance the expression of photoreceptors, or impact the hatchability or livability of the ducklings. We report that in the absence of light during development, the *OPN4M* (melanopsin), a nonvisual sensory receptor, is expressed in the brain embryonically, and that its expression increases prior to hatch. We also determined that the amount of expression of *OPN4M* and retinal cone and rod PRs were not altered in the absence of light during development of the Pekin duck. Thus, the data do not support our hypothesis that photosensory mechanisms were negatively impacted in the absence of light in the Pekin duck. Rather, these data support a novel role for *OPN4M* beyond its known effects on reproduction in adult brains (Scanes et al., 1980; Wilson and Reinert, 1996; Kosonsiriluk et al., 2013; Porter et al., 2018; Potter et al., 2018). To our knowledge, this is the first work to examine photoreceptor and DBP gene expression levels in the central nervous system of a developing embryo incubated in a light or dark environment. Our novel work suggests that incubation in the dark does not negatively affect photoreceptor gene expression levels in the retina. Interestingly, one study found that rearing mice in the dark negatively impacts retinal vasculature during development (Rao et al., 2013). Taken together, our data suggest a role for *OPN4M* in the brain, or retina, during development in both birds and mammals, respectively. In birds, DBPs such as *OPN4M*, are required for seasonal gonadal recrudescence and fertility, and the duck is no exception (Haas et al., 2017; Potter et al., 2018). In the adult, when light is diminished in Pekin duck breeder barns, sex differences in the expression of reproductive behaviors are observed in the Pekin duck, with drakes showing higher sensitivity to diminished light than hens (Porter et al., 2018). *OPN4M* expressing neurons are known to be in the pre-mammillary nucleus of the hypothalamus and to be involved with photoreception and fertility in birds (Scanes et al., 1980; Schew et al., 1996; Li and Kuenzel, 2008; Yamaguchi et al., 2017). In the adult, immunolesions of OPN4-expressing neurons reduces reproductive behaviors and elicits gonadal regression (Potter et al., 2018). These data suggest that the ontogeny of *OPN4M* may not impact the fecundity of the Pekin duck until adolescence or maturity. *OPN4M* expression may be linked to peri-hatch role of thyroid hormone as related to duckling behaviors. It has been shown that DBPs stimulate neurons that signal the mediobasal hypothalamus (**MBH**), which is used monitor day length in order to stimulate gonadal recrudescence. Upon stimulation, cells within the MBH produce type 2 deiodinase (**DIO2**) that converts thyroxine to triiodothyronine. During short day lengths, cells of the MBH produces thyroid hormone-deactivating enzyme that converts thyroxine and triiodothyronine into an inactive form of thyroid hormone (Sirsat and Dzialowski, 2020). Thyroid hormone is also critical for many physiological changes associated with post-hatch development and maturation of the central nervous hormone (**CNS**). Thyroid hormone is also necessary for normal growth and CNS development, as well as the onset of puberty (Sirsat et al., 2018). However, any link between DBPs and thyroid hormone in the developing embryo has yet to be determined in any avian species. Thyroid hormones in birds have numerous functions including weight gain, fattening, and muscle hypertrophy to prepare birds for migration (Pérez et al., 2016). Thyroid hormones are also closely related to the hatching process, particularly in precocial species such as ducks. For example, a larger peri-hatch increase in hypothalamic-pituitary-thyroid axis activity is seen in precocial compared to altricial species (Schew et al., 1996). Increased thyroid hormone levels around hatch have been associated with the critical period of post-hatch imprinting, and exogenous T_3_ at this time can enhance imprinting and learning in chicks (Yamaguchi et al., 2012). Although the relationship of peri-hatch thyroid hormones and imprinting and learning in Pekin ducks is unknown, it has been demonstrated that increased thyroid hormones near the end of incubation are involved in cholinergic and adrenergic mediated regulation of cardiovascular development (Sirsat et al., 2018; Sirsat and Dzialowski, 2020), as well as the initial thermoregulatory responses to cooling at hatch, and ultimately thermoregulation (Spiers et al., 1974; Olson et al., 1999). Thus, the potential link between DBP expression and the hypothalamic-pituitary-thyroid system may have important impacts upon behavior, welfare and physiological function of our domestic avian species beyond the reproductive system. However, our hatchery data show no apparent differences in production or general measures of growth when allowed to develop with or without light. Furthermore, the results suggest no difference in rod and cone photoreceptor gene expression levels in the developing Pekin duck after light treatment.

Several studies have found DBP *OPN4M* in various regions of the adult brain. For example, in the Zebrafish the photoreceptive region within the brain has been pinpointed in the preoptic area (Fernandes et al., 2012). In a study on Teleost fish, findings discovered the thalamus region of the brain to express opsin genes, forms of DBPs (Hang et al., 2016). More relevant to the Pekin duck, DBPs have been found to display gene expression in the septal-hypothalamic regions in chicks (Kuenzel et al., 2015), and specifically melanopsin has been identified in the premammillary nucleus of the hypothalamus in several poultry species including the duck (Spiers et al., 1974; Li and Kuenzel, 2008; Haas et al., 2017). A recent study in the duck has also shown increased expression levels of *OPN4M* at hatch, which then decreases until just prior to the onset of puberty (Van Wyk and Fraley, 2021). Therefore, our study shines some light on the development of the Pekin duck brain in relation to the DBP OPN4M. Our results show a significant interaction and main effect of time of *OPN4M* gene expression during normal development in light. As expected, the later stages of development displayed a greater relative gene expression of *OPN4M*, relative to their earlier stages. However, no comparison can be made between light and dark treatment groups. Future studies examining *OPN4M* and other DBPs are warranted to determine the impact darkness has on DBP function.

Lastly, the ontogeny of *Per2*, a circadian clock gene, in the developing chick embryo increases in expression around ED 16-18 in the brain (Okabayashi et al., 2003). Okabayashi and authors found that a light/dark cycle was necessary to observe Per2 expression. This developmental time course of *Per2* expression also aligns with the increase in OPN4M in our current study. However, in a KO *Opn4* mouse model, there was no impact on circadian entrainment (Panda et al., 2003). Although, Panda and authors do suggest other genes such as cryptochromes may be involved.

## CONCLUSIONS

We have shown that the presence or absence of light may not alter the expression patterns of retinal PRs or DBP *OPN4M* mRNA during pre-hatch development. Further we have shown that the presence of light in the incubators does not affect production variables in the hatchery nor beyond in commercial barns. Interestingly, we show an increase in *OPN4M* mRNA levels at a later pre-hatch developmental time point (ED 21). This result is similar to a previous study that also showed increased *OPN5* levels on the day of hatch (Van Wyk and Fraley, 2021). With brain OPN4M expression occurring close to the hatch day, future studies should investigate the link between *OPN4M* and thyroid hormone actions in the embryonic bird (Van Wyk and Fraley, 2021), as well as to determine the long-term effects of incubator lighting systems on adult behaviors and fertility in breeder ducks.

## Appendix Supplementary materials

Image, application 1

Image, image 2

Image, image 3
