# Supplementary material for: Effect of pre-hatch incubator lights on the ontogeny of CNS opsins and photoreceptors in the Pekin duck
Source: Poult Sci. 2022 Jan 10;101(4):101699. doi: 10.1016/j.psj.2022.101699 (PMC8857459; doi:10.1016/j.psj.2022.101699)
Supplement: Supplementary file 1 [file mmc1.docx]

# Supplemental Figure legends

**Supplemental Figure 1**: Spectrogram

A) Spectrogram of LED (Once Innovation, Inc) lights placed in incubators.

B) For comparison, spectrogram from typical white fluorescent light.

C) Representative light measurements from data logger recorders placed in an egg slot on shelf closest (Close) to LED lights, and one from an egg slot furthest (Far) from the LED lights. Lights off was at 1800hrs and lights on at 0600 hrs. Data loggers recorded the greatest light intensity when egg shelves were oriented towards the lights, and the lowest intensity when shelves are oriented away from LEDs. Data loggers closest to the LEDS showed higher lux measures compared to those further away. On the right side of the graph are the photonic energies associated with different lux levels as measured by spectrophotometry.

**Supplemental Figure 2**: Experimental timeline. Before tissue extraction, samples were incubated in 4 ^o^C for 5 days without light. Tissues were extracted on post-incubation days 3, 7, 11, 16, and 21 are represented by images of embryos (days 3 and 7), retina, and brain (days 11, 16, and 21).
