# Supplementary figures and images for: Effect of pre-hatch incubator lights on the ontogeny of CNS opsins and photoreceptors in the Pekin duck
Source: Poult Sci. 2022 Jan 10;101(4):101699. doi: 10.1016/j.psj.2022.101699 (PMC8857459; doi:10.1016/j.psj.2022.101699)

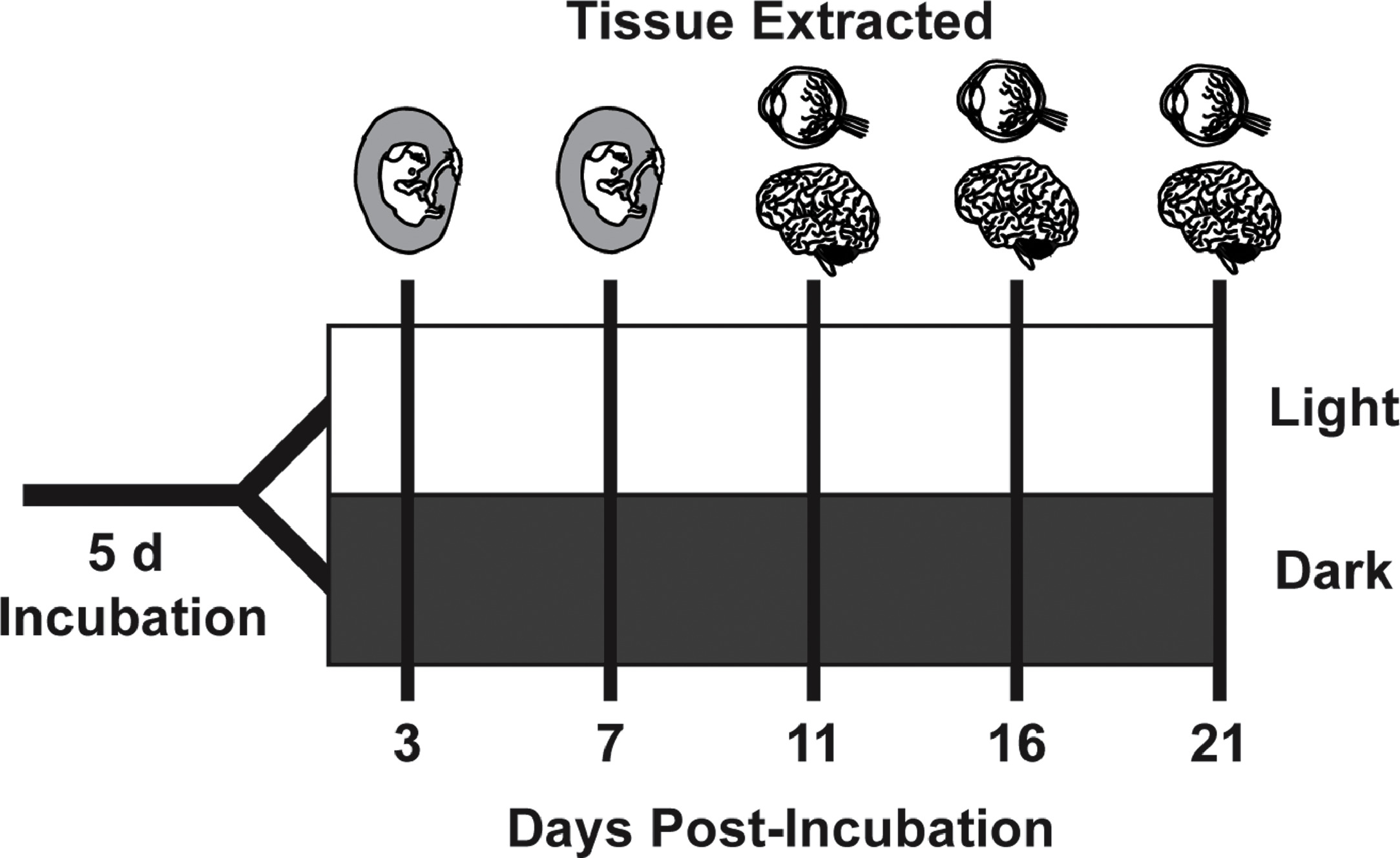

Supplement: Supplementary file 2 [file mmc2.jpg]

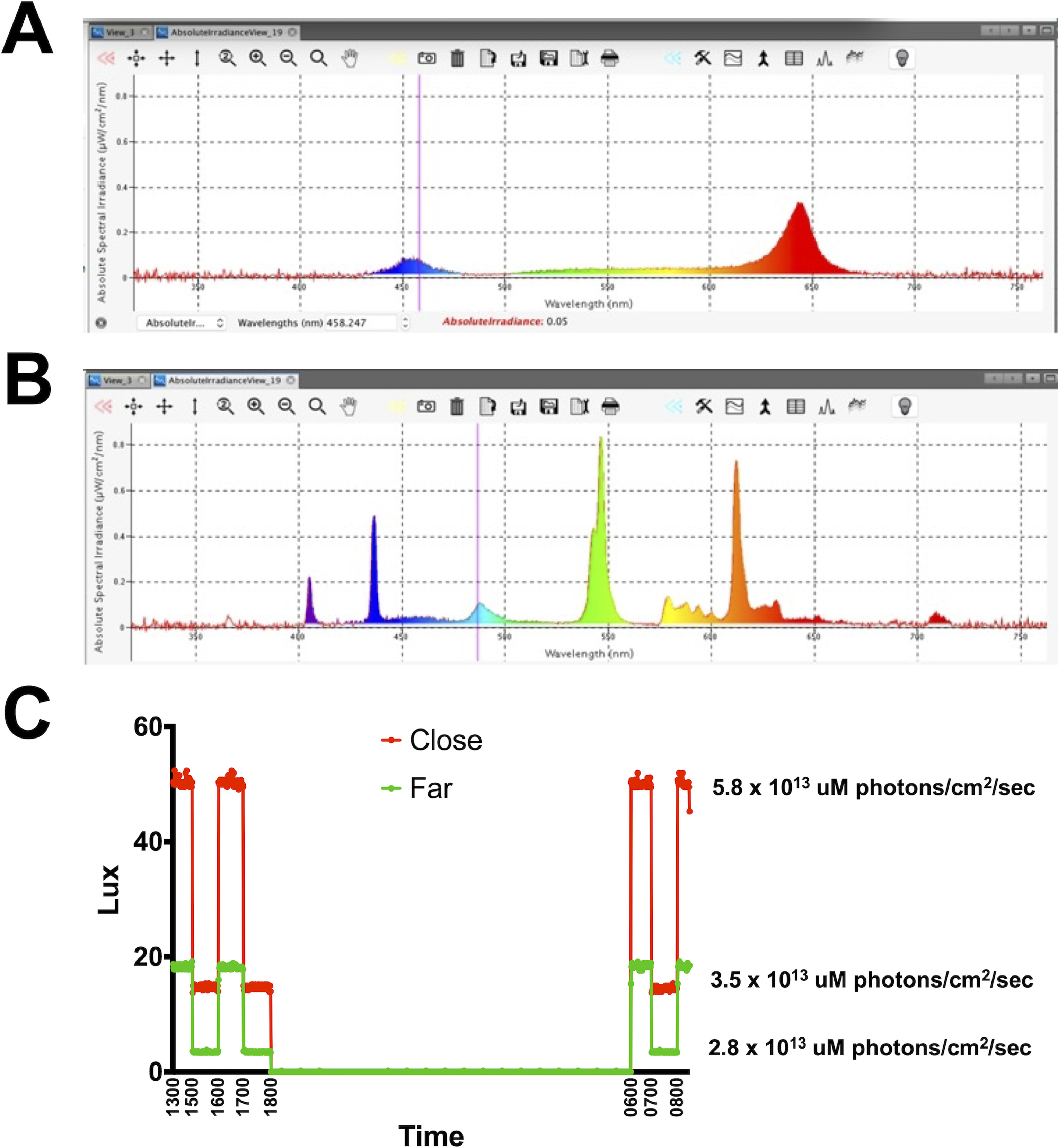

Supplement: Supplementary file 3 [file mmc3.jpg]
